# Novel Insights Into Triple-Negative Breast Cancer Prognosis by Comprehensive Characterization of Aberrant Alternative Splicing

**DOI:** 10.3389/fgene.2020.00534

**Published:** 2020-06-11

**Authors:** Shasha Gong, Zhijian Song, David Spezia-Lindner, Feilong Meng, Tingting Ruan, Guangzhi Ying, Changhong Lai, Qianqian Wu, Yong Liang

**Affiliations:** ^1^Institute of Cancer Research, Department of Basic Medicine, School of Medicine, Taizhou University, Taizhou, China; ^2^Precision Medicine Center, Taizhou University Hospital, Taizhou University, Taizhou, China; ^3^Bio-X Institutes, Key Laboratory for the Genetics of Developmental and Neuropsychiatric Disorders (Ministry of Education), Collaborative Innovation Center for Brain Science, Shanghai Jiao Tong University, Shanghai, China; ^4^School of Medicine, University of Texas Health San Antonio, San Antonio, TX, United States; ^5^Institute of Genetics, Zhejiang University, Hangzhou, China

**Keywords:** alternative splicing, triple-negative breast cancer, The Cancer Genome Atlas, prognostic biomarker, splicing correlation network

## Abstract

**Background:**

Alternative splicing (AS) is important in the regulation of gene expression and aberrant AS is emerging as a major factor in the pathogenesis of human conditions, including cancer. Triple-negative breast cancer (TNBC) is the most challenging subtype of breast cancer with strong invasion, high rate of metastasis, and poor prognosis. Here we report a systematic profiling of aberrant AS in TNBC.

**Methods:**

The percent spliced in (PSI) values for AS events in 151 TNBC patients were obtained from The Cancer Genome Atlas (TCGA) SpliceSeq database. Univariate Cox and stepwise Multivariate Cox regression analyses were conducted to find the best prognostic AS model. Splicing regulatory networks were constructed by prognosis-related spliceosome and aberrant AS events. Additionally, pathway enrichment and gene set enrichment analysis (GSEA) were further employed to reveal the significant pathways for prognosis-related AS genes. Finally, splicing regulatory networks were constructed via Spearman’s rank correlation coefficients between prognosis-related AS events and splicing factor expressions.

**Results:**

A total of 1,397 prognosis-associated AS events were identified in TNBC. The majority of the parent genes of prognostic AS events exhibited direct interactions to each other in the STRING gene network. Pathways of focal adhesion (*p* < 0.001), RNA splicing (*p* = 0.007), homologous recombination (*p* = 0.042) and ECM-receptor interaction (*p* = 0.046) were found to be significantly enriched for prognosis-related AS. Additionally, the area under curve (AUC) of the best AS prognostic predictor model reached 0.949, showing a powerful capability to predict outcomes. The Exon Skip (ES) type of AS events displayed more robust and efficient capacity in predicting performance than any other specific AS events type in terms of prognosis. The ES AS signature might confer a strong oncogenic phenotype in the high-risk group with elevated activities in cell cycle and SUMOylating pathways of tumorigenesis, while programmed cell death and metabolism pathways were found to be enriched in the low-risk group of TNBC. The splicing correlation network also revealed a regulatory mode of prognostic splicing factors (SFs) in TNBC.

**Conclusion:**

Our analysis of AS events in TNBC could not only contribute to elucidating the tumorigenesis mechanism of AS but also provide clues to uncovering underlying prognostic biomarkers and therapeutic targets for further study.

## Introduction

Breast cancer is one of the most lethal malignancies in women worldwide with an estimated 268,600 new cases diagnosed per year in the United States (DeSantis et al., 2019). From a pathological viewpoint, breast cancer can be characterized into four basic subtypes with the presence or absence of two hormone receptors including estrogen receptor (ER), progesterone receptor (PR), and human epidermal growth factor receptor 2 (HER2) (Onitilo et al., 2009; Dai et al., 2016). TNBC is defined as the lack of ER, PR and the absence of protein overexpression/gene amplification of HER2, which accounts for approximately 15 to 20% of all breast cancers (Iorio et al., 2016). Compared with other subtypes, TNBC is the most clinically challenging subtype due to its strong invasion, high rate of metastasis, and poor OS. To date, chemotherapy remains the only standard treatment, while other therapies did not exhibit any significantly improvement of survival for TNBC (Bianchini et al., 2016). Furthermore, TNBC is a highly heterogeneous disease whose molecular mechanism of progression and aggressiveness is still unclear. Therefore, exploration of the mechanism that promotes TNBC progression and development of novel predictive prognostic biomarkers are crucial.

Over the last few decades, major efforts have been made to reveal the underlying mechanisms of TNBC that could assist in the prediction of prognostic biomarkers and the guidance of anti-cancer treatments. In addition, extensive studies have been performed on genomic, transcriptomic, and even DNA methylation features to classify TNBC into intrinsic molecular subtypes, which have widely expanded our knowledge on TNBC (Bianchini et al., 2016; Stirzaker et al., 2016; Shi et al., 2018). However, these studies only focus on mutation and abnormal gene expression while ignoring other important oncogenesis mechanisms such as aberrant alternative splicing.

**FIGURE 1:**
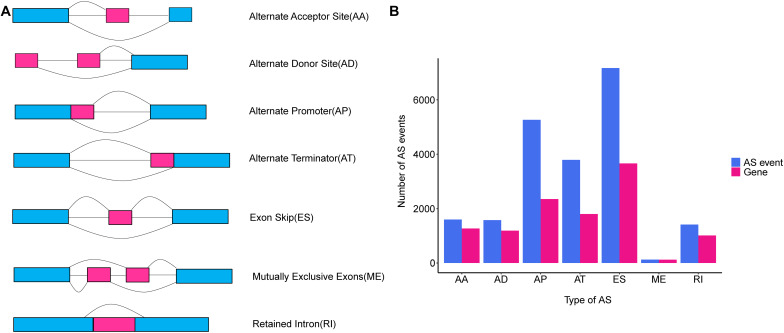
An overview of seven classical subgroups of alternative splicing (AS) patterns. **(A)** Schematic illustration splicing pattern of seven subgroups of AS events. AA, alternate acceptor site; AD, alternate donor site; AP, alternate promoter; AT, alternate terminator; ES, exon skip; ME, mutually exclusive exons; RI, retained intron. **(B)** The number of AS events (blue bars) and genes in parents (red bars) among the seven subgroups of TNBC taken from analysis of 151 TNBC patients in TCGA SpliceSeq database.

Alternative splicing is a post-transcriptional modification mechanism to ensure high transcript and protein diversity during the lifespan of eukaryotic organisms (Wang et al., 2015). It has been estimated that up to 95% of multi-exon genes undergo the AS process, and the vast majority of them vary across different human cells and tissues (Pan et al., 2008; Wang et al., 2008; Ke and Chasin, 2011). AS events are categorized into seven common types as follows: Alternate Acceptor site (AA); Alternate Donor site (AD); Alternate Promoter (AP); Alternate Terminator (AT); Exon Skip (ES); Mutually Exclusive Exons (ME), and RI (Figure 1A) (Ryan et al., 2012, 2016). Growing evidence indicated that the aberrant AS patterns were implicated in many human cancers (Oltean and Bates, 2014). Deep analyses of AS events in non-small cell lung cancer, gastrointestinal cancer and breast cancer have provided abundant clues linking aberrant AS events and the tumorigenesis of cancer (Li et al., 2017; Lin et al., 2018; Zhang et al., 2019). Particularly, global dysregulation of AS was regarded as a driver event causing breast cancer progression and metastasis (Read and Natrajan, 2018). Despite this, the underlying regulatory mechanisms of AS and their clinical implications were not yet fully understood in TNBC. Hence, identification of aberrant AS events as a potential prognostic biomarker and therapeutic target for TNBC is both promising and imperative.

In the present study, we conducted a comprehensive analysis of genome-wide AS events in the TNBC from TCGA. Subsequently, we determined prognosis-associated AS events and uncovered reliable and distinct prognostic signatures for TNBC. In addition, we constructed a prognosis-associated splicing factor-alternative splicing (SF-AS) network to explore the splicing factors and their potential targets. Our findings represent an initial attempt to uncover TNBC related AS events. Moreover, further exploration of splicing regulatory factors on their AS targets may aid in fully understanding the underlying mechanism of splicing factors, which could eventually help to elucidate the role that AS plays in the tumorigenesis of TNBC.

## Materials and Methods

### TCGA RNAseq and Alternative Splicing Event Data Process

RNAseq-HTSeq data and clinical data of patients who were diagnosed with primary breast cancer were downloaded from the TCGA^1^. The inclusive criteria for categorizing TNBC are as follows: (1) loss of expression for ER and PR, and HER2 by immunohistochemistry (IHC), and the absence of the amplification of HER2 by fluorescent *in situ* hybridization (FISH) being needed if the IHC result was equivocal (Penault-Llorca and Viale, 2012); (2) patients with clinicopathological information and who had an OS of at least 30 days; (3) patients did not suffer from other types of tumors. Based on these screening criteria, a total of 151 TNBC samples were enrolled in this study (Penault-Llorca and Viale, 2012).

AS profilings of TNBC cohorts were downloaded from TCGA SpliceSeq^2^ (Ryan et al., 2016), a web-based resource for generating splicing patterns of TCGA tumors. Percent-splice-in (PSI) value is used to quantify splicing events, it represents the ratio of inclusive reads over inclusive and exclusive reads normalized by read length in each splicing event, and always ranges from 0 to 1. The inclusion criteria for a reliable set of AS events were required the percentage of Samples with PSI Value ≥ 75% and a standard deviation of > 0.05. In addition, missing splicing data were imputated by the K-nearest neighbor (KNN) algorithm from Pamr package in R.

### UpSet Plot Functional and Pathway Enrichment and PPI Networks Analysis

Given that one gene harbors more than one type of AS events, intersections between protein-coding genes in seven types of AS events in TNBC were visualized by UpsetR package in R. The GO (Ashburner et al., 2000) of BP, CC, MF, and KEGG pathway (Kanehisa et al., 2017) were analyzed by the DAVID (version 6.8) (Huang da et al., 2009) web tool with default settings, and was visualized by the gglpot2 package in R. Subsequently, the parent genes of the most prognostically significant AS events (*p* < 0.001) were imported into STRING database (version 11) (Szklarczyk et al., 2019) with a confident score >0.4 to construct a gene-interaction network. Cytoscape (version 3.7.1) (Shannon et al., 2003) was further applied to display their relationships in the gene-interaction network.

### Survival Analysis and the Construction of Prognostic Model

For each type of AS events, TNBC cohorts were divided into two groups by a median cut of PSI value. Univariate Cox proportional hazard regression analysis was employed to detect the survival-related AS events with *p* < 0.05 in all seven AS subgroups. The most significant top 10 genes in each AS event model were chosen as candidates for the forest plots. The top 10 prognostically aberrant AS gene were then merged into a Multivariate Cox regression model to build candidate prognostic models. To make our model more practical and robust, a forward stepwise method was used to search a minimal set of candidate AS events. In addition, a combination of AS PSI levels weighted by regression coefficient (β) from the Multivariate Cox regression analysis was used to construct a risk score model (Scosyrev and Glimm, 2019). The risk score for each patient was as follows: Risk score = β_*1*_ × AS_1_ + β_*2*_ × AS_2_ + ⋯ + β_*n*_ × AS_*n*_. Kaplan–Meier Curves (Logrank Tests) were used to verify whether the predictive models could differentiate the OS between high-risk and low-risk groups in TNBC. The AUC of the Receiver Operating Characteristic (ROC) curve was calculated by survival ROC package for each AS events based on the prognostic model. All statistical analyses were performed using R (version 3.5.3).

### Gene Set Enrichment Analysis (GSEA) for the AS Event-Based Classifier

To further explore the biological pathways in the AS Event-Based predictive model, GSEA analysis (Subramanian et al., 2005) was performed to verify the differences in BP and pathways between high-risk and low-risk groups with the JAVA program using the annotated gene set of “C2: Canonical pathways” which was downloaded from the MSigDB^3^. After performing 1000 permutation steps, the gene sets with nominal *p* < 0.05 were considered to be significantly enriched.

### Construction of the Splicing Factors (SFs) Correlation Networks

The associations between prognostic AS events and their regulated SFs were further investigated. A list of 71 known SFs was extracted from the SpliceAid2 database^4^ (Piva et al., 2012). The curated level 3 mRNA-seq data of SFs were downloaded from TCGA portal^1^ and normalized by TMM method in edgeR packages (Robinson et al., 2010). To find a potential regulatory network between the prognosis-associated SFs and AS events, Univariate Cox regression analysis was first conducted to screen survival-associated SFs, and then Spearman test was implemented to analyze the correlation between the mRNA expression level of prognostic SFs and PSI values of AS events. The significantly correlated pairs for SFs with PSI values of AS events (|r| > 0.4, *p* < 0.001) were selected as the candidates for the splicing correlation network. Lastly, Cytoscape (version 3.7.1) was further used to plot the potential SF-AS regulatory network of the significant correlation between SFs and AS events.

## Results

### Integrated Overview of AS Events Profiling in TNBC Cohort

The comprehensive genome-wide AS events profiling of 151 TNBC patients with clinical implications was generated by TCGA SpliceSeq, in among which the median follow-up OS was 30.5 months (range 1 to 117 months). To give a precise description for each AS event, taking “HNRNPA1_ES_212638” for example, “HNRNPA1” represented the parent gene of the AS event, “ES” was the AS type, and the number “212638” stood for the AS event ID from the TCGA SpliceSeq database. Totally, we detected 20,931 AS events from 7,358 genes, including 7,166 ESs in 3,661 genes, 1,413 RIs in 1012 genes, 5,263 APs in 2,353 genes, 3,789 ATs in 1,802 genes, 1,575 ADs in 1,189 genes, 1,600 AAs in 1,269 genes and 125 MEs in 122 genes (Figure 1B). Additionally, it should be noted that one gene harbored up to three AS events on average. Among these genes, Collagen Type I Alpha 2 Chain (COL1A2) had the max number of AS events (*n* = 48), followed by Mitochondrial Ribosomal Protein L55 (MRPL55) (*n* = 45) and Arylformamidase (AFMID) (*n* = 36), which suggested that one gene with multiple AS events could result in combinatorial arrangements and greatly contribute to transcriptome and proteome diversity. Among these splicing patterns, ES events were observed as the most frequent type, which accounted for 34.2% of the AS types, followed by AP (25.1%) and AT (18.1%) events.

### Identification of Prognosis-Associated AS Events in TNBC

**FIGURE 2:**
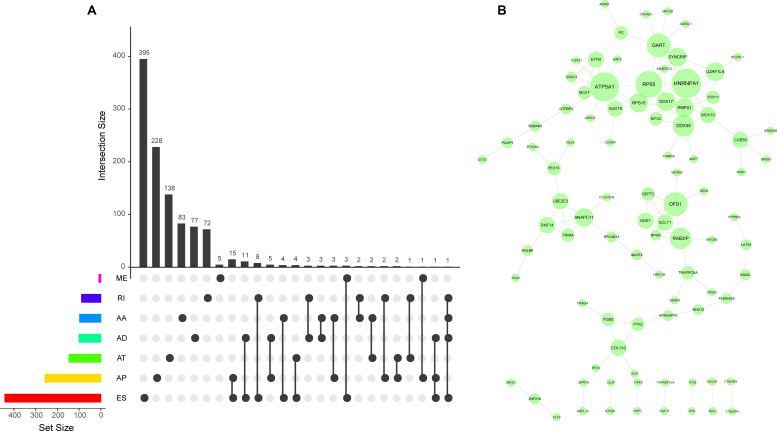
UpSet and gene interaction network plots of prognosis-associated AS events in TNBC. **(A)** The UpSet intersection plot between the seven types of prognosis-associated AS events and genes. One gene may have up to four types of AS events. **(B)** Gene interaction network for prognosis-associated AS events. This plot was generated by cytoscape, nodes stood for genes, lines denoted interactions, and the size of the nodes represented degrees.

To explore the association between AS events and prognosis in patients with TNBC, we conducted cox univariate analyses to evaluate the prognostic factor in each AS event type. TNBC patients were categorized into low- and high-PSI groups according to the median PSI value. Consequently, a total of 1,397 AS events were found to be significantly associated with OS (log-rank *p* < 0.05), which accounted for 6.7% of all AS events and 14.5% of all the patent genes in TNBC (Supplementary Table 1). It was noteworthy that most of these significant prognosis-associated AS events (800 ASs out of 1,397 ASs) were favorable prognostic factors (HR < 1). To quantitatively analyze the overlap between genes and AS events amongst the seven AS types, the UpSet plot was created to visualize the intersecting sets in Figure 2A, indicating that most of the prognosis-associated AS genes might have one AS events type, and some of them may have up to four AS event types. For example, AA, AD, ES, and RI events of TMEM205 were found to be significantly associated with TNBC patients’ survival.

### Protein–Protein Network Analyses of Survival-Associated AS Events

To investigate the interactions among these genes of prognosis-associated AS events, we conducted gene interaction network analyses in TNBC. Parent genes of prognosis-associated AS events (*p* < 0.001) were imported to the STRING PPI database (version 11) with a score > 0.4. A PPI-network generated by cytoscape illustrated the hub genes (HNRNPA1, RPS5, DDX55, OFD1, et al.) and their interactions in the prognosis related network (Figure 2B). Our results suggested that the majority of the parent genes of prognostic AS events exhibited direct gene–gene interaction to each other in the STRING gene network, indicating AS dysregulation genes played a crucial role in the process of TNBC tumorigenesis.

### Functional Enrichment Analysis of Survival-Associated AS Events

**FIGURE 3:**
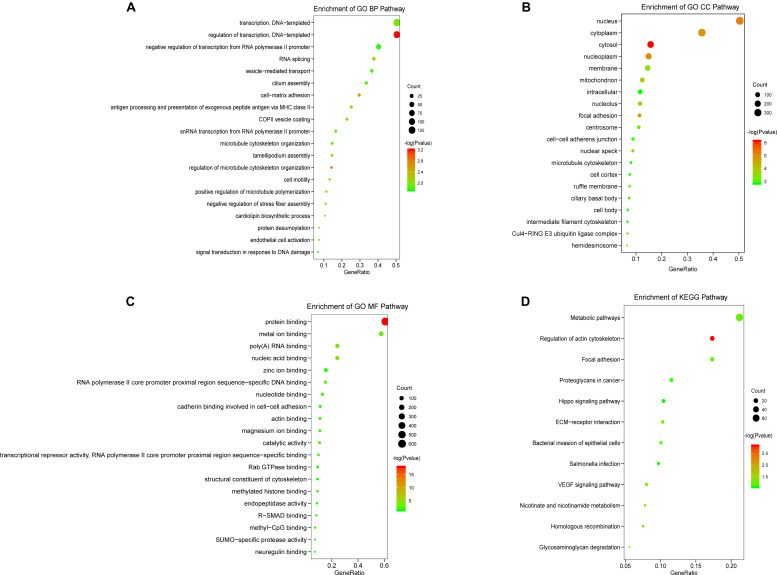
KEGG and GO pathways for TNBC prognosis-associated AS genes. The vertical axis represents GO or KEGG pathway annotations. The horizontal axis represents the number of genes assigned to the corresponding annotated pathway. **(A)** The top 20 significant terms of BP in GO. **(B)** The top 20 significant terms of CC in GO. **(C)** The top 20 significant terms of MF in GO. **(D)** The top 12 terms of KEGG pathways.

Next, in order to evaluate the potential impact of prognosis-associated AS events on corresponding gene biological functions in TNBC, GO, and KEGG pathways enrichment analyses for their parent genes were performed. The results revealed that a total of 49 significant terms in GO BP categories were closely related to TNBC, including regulation of transcription, DNA-templated (*p* < 0.001), and RNA splicing (*p* = 0.007) (Figure 3A). Besides, 29 pathways in GO CC indicated significant differences in terms such as focal adhesion (*p* < 0.001) and Cul4-RING E3 ubiquitin ligase complex (*p* = 0.0015) (Figure 3B). We also observed that 24 pathways in GO MF (Figure 3C) were highlighted, including significant difference in terms of poly(A) RNA binding (*p* < 0.001), RNA polymerase II core promoter proximal region sequence-specific DNA binding (*p* = 0.0016), as well as SUMO-specific protease activity (*p* = 0.005). Additionally, 5 KEGG pathways associated with TNBC carcinogenesis were significantly affected in TNBC (*p* < 0.05, Figure 3D), including Regulation of actin cytoskeleton (*p* = 0.0015), Homologous recombination (*p* = 0.042) and ECM-receptor interaction (*p* = 0.046). Enriched KEGG pathways were depicted in Figure 3D, and the detailed significant pathway enrichment results were listed in Supplementary Table 2. Collectively, these findings suggested that the prognostic associated AS events not only played a vital role in tumor proliferation pathways such as Homologous recombination and RNA splicing, but also participated in invasion and metastasis related processes such as focal adhesion and ECM-receptor interaction, which could contribute to uncovering the underlying mechanisms linking OS-associated AS events and protein biological function during the carcinogenesis in TNBC.

### Construction of the Prognostic Predictors in TNBC Cohort

**FIGURE 4:**
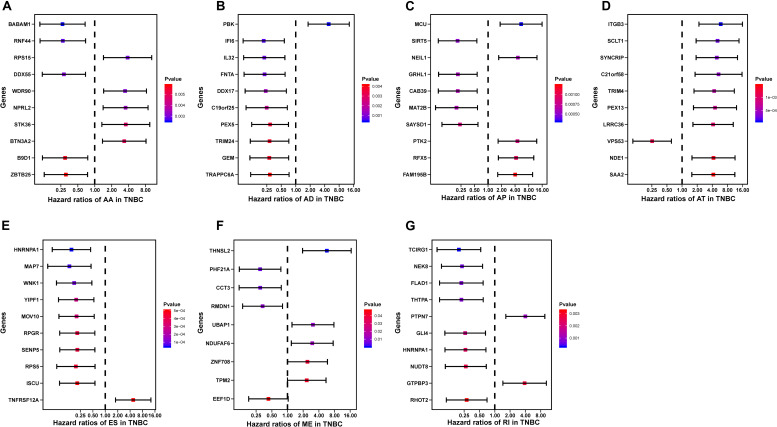
The forest plots of the top 10 prognosis-associated AS events in TNBC. **(A–G)** HR of the top 10 prognosis associated AS parent genes, including AA subgroup **(A)**, AD subgroup **(B)**, AP subgroup **(C)**, AT subgroup **(D)**, ES subgroup **(E)**, ME subgroup **(F)**, and RI subgroup **(G)** events. The Circles represent HR and Horizontal bars represent 95% CIs. *p*-Values of univariate Cox analyses are indicated by color scale on the right side.

**FIGURE 5:**
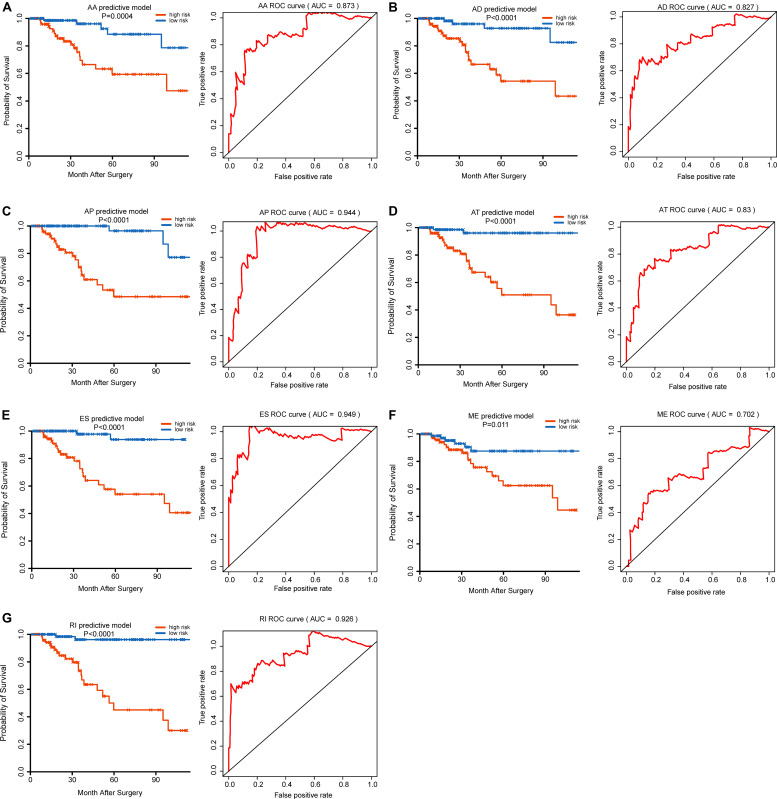
Kaplan–Meier (K–M) plot and time-dependent ROC curves for the multivariate prognostic AS models in TNBC. Kaplan–Meier curves of the multivariate prognostic models were generated for **(A)** AA, **(B)** AD, **(C)** AP, **(D)** AT, **(E)** ES, **(F)** ME, and **(G)** RI patterns of AS, respectively. In K–M plot, red line represents high-risk subgroup while blue line represents low-risk subgroup. ROC curves with AUC for 3-year survival were constructed with each type of AS event in TNBC. AUC value of the ROC curve of >0.7 was observed in each type of AS in TNBC.

**FIGURE 6:**
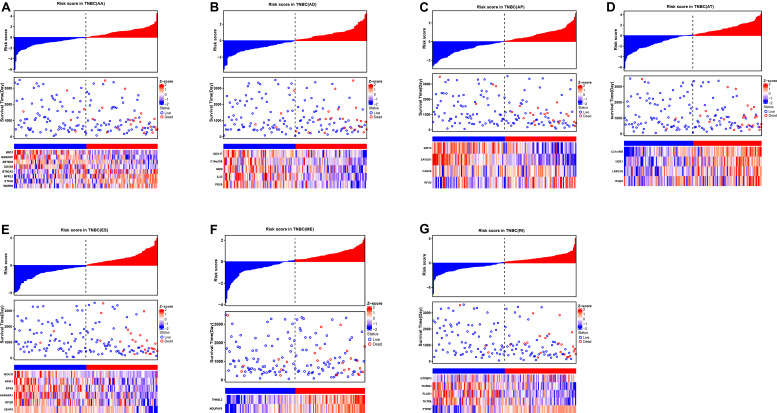
The prognostic AS signatures for TNBC patients in multivariate Cox regression analysis. TNBC patients were divided into low- and high-risk groups according to by the median value of risk score. Risk scores were built for **(A)** AA, **(B)** AD, **(C)** AP, **(D)** AT, **(E)** ES, **(F)** ME, and **(G)** RI of prognosis-associated splicing events. In each subplot, the top part of the integrated graph represents risk score distribution curves, the middle part exhibit the survival time and status distributed by based on risk score, and the bottom part shows the heatmap of the normalized PSI index for each AS prognostic signature. Color from blue to red indicates the increasing z-score of PSI values from low to high.

To evaluate the independent prognostic factors of AS events in the TNBC cohort, the top 10 significant prognostic related AS events were chosen as candidates, which could be displayed in forest plots (Figure 4). Multivariate Cox regression analysis for independent prognostic factors was subsequently employed to construct prognostic predictor models in seven AS events types, respectively. Risk score was first calculated in each AS events type, and the TNBC patients were stratified into high- and low-risk subgroups by the median value of risk scores. In our analysis of each splicing prognostic signature, the prognostic models built with seven different AS types 8 AA events, 5 AD events, 4 AP events, 4 AT events, 6 ES events, 2 MEs or 5 RI events, showed great powers (*p* < 0.0001) to predict the prognostic outcome respectively (Figure 5). Particularly the prognostic predictor of single ES events type exhibited the most powerful capability to predict the outcome amongst the seven prognostic models. Moreover, time dependent ROC curves from 3 years survival were also employed to compare the efficiency among AS signatures. As shown in Figure 5, an AUC value of > 0.7 was observed in each type of AS in TNBC, and the ES prognostic model exhibited the best predictor efficiency at distinguishing favorable or adverse outcomes (AUC = 0.949), followed by the AP models and RI models with AUCs of 0.944 and 0.926 respectively. The detailed information of these prognosis-associated AS events was summarized in Supplementary Table 3. Additionally, the distribution of survival status, risk score, and the heatmaps for the seven AS splicing patterns in TNBC patients were shown in Figure 6. It could be confirmed that the ES events type displayed a more robust and efficient capacity in predicting performance than any other specific AS events type in aspects of prognosis.

### Gene Set Enrichment Analysis (GSEA) of the ES AS Signature

**FIGURE 7:**
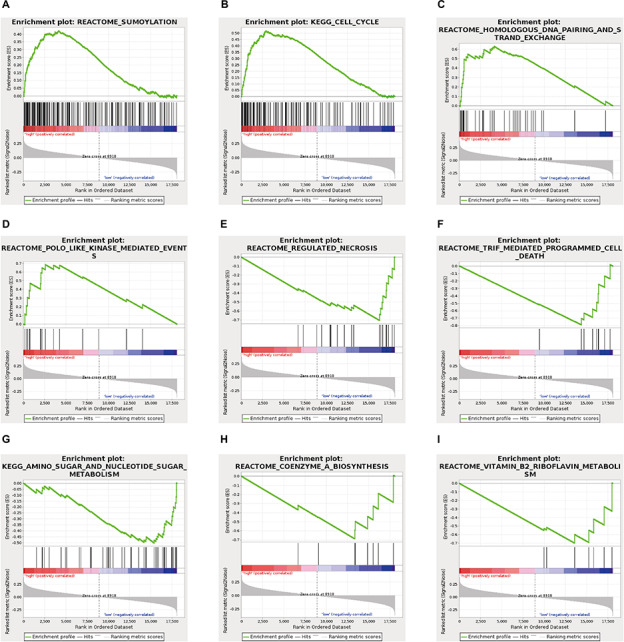
Gene set enrichment analysis analysis delineates biological pathways correlated with the final ES AS event-based classifier using. Hallmark gene sets in “C2: Canonical pathways” section of MSigDB database. The TNBC cohort was classified into low- and high-risk subgroups by ES AS pattern. Significantly enrichment results from GSEA were refer to “SUMOylating,” “Cell cycle,” “Homologous DNA pairing and strand exchange,” POLO like kinase mediated events,” “Regulated necrosis,” “TRIF mediated programmed cell death,” “Amino sugar and nucleotide sugar metabolism,” “Coenzyme A biosynthesis,” and “Vitamin B2 riboflavin metabolism” (**A–I**, see detailed information in Supplementary Table 4).

To explore the specific pathways involved in the ES prognosis-associated AS signature, GSEA analyses were further implemented. By using GSEA with expression of mRNA between low- and high-risk groups from the ES AS signature, several oncogenic pathways were significantly enriched in the high-risk group, including Sumoylation (*p* = 0.01), Cell cycle (*p* = 0.018), Homologous DNA pairing and strand exchange (*p* = 0.022), POLO like kinase mediated events (*p* = 0.035) (Figure 7), which exhibited that they were involved in the tumorigenesis and proliferation of TNBC. On the contrary, specific genes overexpressed in the low-risk group showed significant enrichment in Regulated necrosis (*p* = 0.002), TRIF mediated programmed cell death (*p* = 0.006), Amino sugar and nucleotide sugar metabolism (*p* = 0.02), Coenzyme A biosynthesis (*p* = 0.021), and Vitamin B2 riboflavin metabolism (*p* = 0.025). Detailed GSEA results were listed in Supplementary Table 4. Overall, the ES AS signature might confer a strong oncogenic phenotype in the high-risk group with elevated activities in cell cycle and SUMOylating pathways of tumorigenesis while programmed cell death and metabolism pathways were found to be enriched in the low-risk group of TNBC.

### Construction of a Potential SF-AS Regulatory Network

**FIGURE 8:**
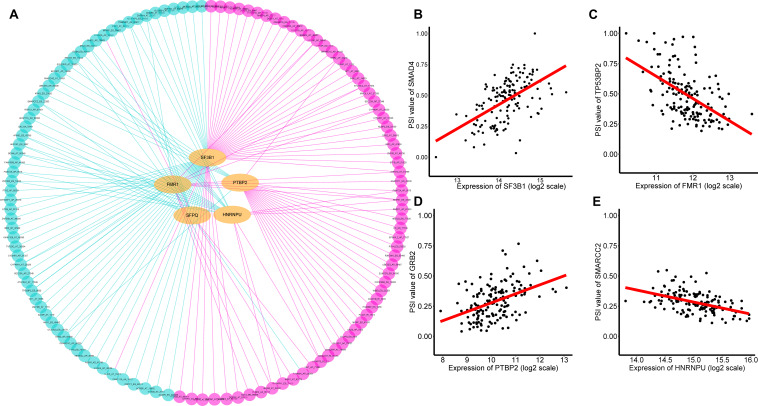
Prognosis related SFs and the splicing regulatory network of TNBC. **(A)** Splicing regulatory network of TNBC was constructed built by Cytoscape software. Splice factors (orange dots) were positively (pink edges) or negatively (green edges) associated with AS events, which predicted favorable (pink dots, HR < 1) or adverse (green dots, HR > 1) outcomes in patients with TNBC. **(B–E)** A correlation between the expression of AS factor and the PSI value of prognosis-associated splicing event.

It is widely acknowledged that several cancer spliceosomes are involved in the regulation of AS events during tumor progress (El Marabti and Younis, 2018), therefore construction of the SF-AS regulatory network based on the significant correlations between prognosis-associated SFs and AS events was necessary. Herein, a total of 71 experimentally validated SFs from the SpliceAid2 database were selected to identify prognosis-related splicing factors. We detected 5 key SFs whose expression levels were strongly associated with prognosis (*p* < 0.01) in TNBC, these are Heterogeneous Nuclear Ribonucleoprotein U (HNRNPU), Splicing Factor 3b Subunit 1 (SF3B1), FMRP Translational Regulator 1 (FMR1), Polypyrimidine Tract Binding Protein 2 (PTBP2), and Splicing Factor Proline and Glutamine Rich (SFPQ). Furthermore, spearman correlation coefficient was used to assess the association between prognosis-associated SFs and AS events. After that, we characterized the SF-AS regulatory network by five prognostic splicing factors and the most significant survival-associated AS events (*p* < 0.001) in TNBC (Supplementary Table 5). As depicted in the SF-AS regulatory network (Figure 8A), it was observed that most favorable survival-related AS events (pink dots) were positively regulated (pink edges) with the expression of SFs while the majority of adverse prognostic AS events (green dots) were negatively (green edges) correlated by SFs, which was consistent with results of survival-associated SFs. Notably, the majority of key AS factors (orange dots) were observed to be related to more than one AS event, and some of them even played opposite roles in regulation of different AS events. Moreover, we also observed that different SFs could affect the same AS event simultaneously, for example, splicing factors SFPQ and HNRNPU were both significantly associated with AA of QKI, which implied that splicing process of the same gene might be co-regulated by different prognostic SFs. Representative correlations between SFs and their survival-related AS events were displayed in the scatter plots (Figures 7B–E). For example, expression of SF3B1 was positively correlated with AP of SMAD4 with *r* = 0.55, *p* < 0.001 (Figure 8B), whereas FMR1 negatively correlated with ES of TP53BP2 with *r* = −0.53, *p* < 0.001 (Figure 8C).

## Discussion

Alternative splicing is regarded as one of the important gene regulatory processes that could enable one single gene to generate multiple mRNAs and translated various proteome in human genome. AS has a tremendous impact on processes of cell growth and development, and accumulating investigations suggest that AS perturbation engaged in multiple tumorigenesis processes such as cancer onset, progression, metastasis, hypoxia, as well as angiogenesis, particularly dysregulated in colorectal adenocarcinoma and lung adenocarcinoma (Misquitta-Ali et al., 2011; Lokody, 2014). Carcinogenesis in TNBC is a complex process that involves aberrant mRNA AS and the production of protein diversity. Previous studies demonstrated that the AS events or regulator factors in breast cancer were aberrantly expressed, thus contributing to the pathogenesis mechanisms of tumor formation (Read and Natrajan, 2018). For example, Han et al. (2017) discovered that intron retention was the most dominant type of hypoxia-induced AS in breast cancer. Ke et al. (2018) identified TDP43 as a crucial splicing regulator that interacted with SRSF3, which was responsible for the unique AS profile in TNBC. Nevertheless, these studies were based solely at the level of single genes, while a systematic analysis at the level of the whole genome that could better characterize aberrant AS events as potential prognostic and predictive biomarkers in TNBC is still lacking.

In our study, we downloaded RNA splicing data of 151 TNBC samples from TCGA SpliceSeq database and performed a genome-wide profiling analysis to elucidate the oncogenic roles of AS in TNBC. Our analysis preliminarily detected 1,397 AS events from 1,069 parent genes that were significantly associated with OS in TNBC. According to our results, ES events were observed as the most frequent type, accounting for nearly one third of the AS types, followed by AP and AT events, which was concordant with other studies (Xiong et al., 2018; Li et al., 2019; Zhang et al., 2019). Notably, among the genes with multiple AS events, Collagen Type I Alpha 2 Chain (COL1A2), which is a subunit of type I collagen, has the maximum number of AS events (*n* = 48). In addition, further analysis also identified COL1A2 AS events as being significantly associated with survival. Evidence showed that COL1A2 could activate β1-integrin and the activation, along with the epithelial-mesenchymal transition, participated in the development of various human malignancies (Yu et al., 2018).

From the gene-interaction network analysis, we found that the genes with survival-associated AS events including HNRNPA1, RPS5, DDX55, and OFD1 were hub nodes of the network which might play vital roles in cancer progress. Recent studies have suggested that that HNRNPA1 is involved in the progression and metastasis of several cancers (Chen et al., 2018), including lung, stomach, prostate, and breast cancers (Nadiminty et al., 2015). HNRNPA1 is also a member of the hnRNP family of RNA-binding proteins, which are involved in RNA maturation and translation, as well as pre-mRNA AS (Yang et al., 2019). However, the exact role of AS events in Heterogeneous nuclear ribonucleoprotein A1(HNRNPA1) and its molecular mechanism remains unknown. The hub genes (including HNRNPA1) identified in our gene-interaction network could help to find new clues for the tumorigenesis mechanism of TNBC. GO and KEGG pathway analysis using DAVID were performed to investigate the underlying biological functions for prognosis-associated AS events in TNBC. GO pathway analysis revealed that the genes of prognosis-associated AS events were enriched in focal adhesion, Cul4-RING E3 ubiquitin ligase complex, RNA splicing, and poly(A) RNA binding pathways, and some of which were referred to as the regulator of RNA splicing process (Zhang and Manley, 2013; El Marabti and Younis, 2018). Furthermore, the KEGG enrichment analysis brought us new insights for elucidating hallmark cancer pathways of TNBC, including Regulation of actin cytoskeleton, Homologous recombination and ECM-receptor interaction which play crucial roles in oncogenesis processes (Wallace et al., 2012). For example, lack of focal adhesion and corruption of ECM-receptor interactions has been regarded as a vital characteristic in most cancers. Additional research concerning how these AS events induced aberrant activation of tumor signaling pathways could be explored in the future.

Recently, a prognostic model based on mRNA-lncRNA signature including three mRNA (FCGR1A, RSAD2, CHRDL1), and two lncRNAs (HIF1A-AS2 and AK124454), was constructed to assess the survival and prognosis of TNBC patients (Jiang et al., 2016). However, a prognostic model based on AS events has not yet been reported. Therefore, further analysis on the prognostic models built by single types of seven AS patterns (AA, AD, AP, AT, ES, ME, and RI) was conducted to evaluate the prognosis. The results showed that the prognostic predictor of the ES type exhibited better efficiency to predict the outcome than the other six types of AS prognostic models. In addition, The AUC of ROC from 3 years survival for this high-efficiency prognostic model reach 0.949, providing a more accurate prediction in risk stratification for TNBC patients. GSEA analyses were further employed to reveal specific pathways involved in the ES AS signature between high- and low-risk groups. Notably, we identified genes in the high-risk group that displayed significant enrichment in cell cycle and Sumoylation pathways while the genes in low-risk group exhibited significant enrichment in programmed cell death, and metabolism pathways. Taken together, this research helps us to investigate prognostic AS patterns of the TNBC cohort, which could potentially lead to the effective use of prognostic biomarkers in TNBC.

Another notable finding was the crucial roles of the correlation network between prognostic splicing factors and the significant prognostic-related AS events. In this study, we obtained five splicing factors that were closely associated with the OS of TNBC patients (*p* < 0.01), including Heterogeneous Nuclear Ribonucleoprotein U (HNRNPU), Splicing Factor 3b Subunit 1 (SF3B1), FMRP Translational Regulator 1 (FMR1), Polypyrimidine Tract Binding Protein 2 (PTBP2), and Splicing Factor Proline and Glutamine Rich (SFPQ). Moreover, we observed that expression of PTBP2 had a high correlation with GRB2 AP (*r* = 0.44, *p* < 0.001, Figure 8D). Recent studies reported that GRB2 acts as an adaptor protein which plays a central role in the regulation of ARF1 and ARF6 activation in invasive breast cancer (Haines et al., 2014). Additionally, we also found that HNRNPU had significant correlation with SMARCC2 ES (*r* = −0.49, *p* < 0.001, Figure 8E). SMARCC2, encoding subunits of Mammalian SWI/SNF chromatin remodeling complexes, is thought to be a core factor in chromosomal rearrangements of chromatin regulators (Kadoch and Crabtree, 2015). Recent studies suggested that abnormalities in SWI/SNF complex subunits play a crucial role in breast cancer (Ribeiro-Silva et al., 2019). Our SF-AS network would further strengthen the association of aberrant AS of ES in SMARCC2 with HNRNPU, which could provide invaluable clues to uncover potential AS prognostic biomarker for TNBC. Collectively, our findings present a better understanding of the underlying mechanism of splicing factors, which could eventually help to elucidate the pathogenic role of AS in TNBC.

## Conclusion

In summary, we have provided a comprehensive analysis of aberrant AS events in TNBC. Our study constructed an ES AS signature to evaluate the prognostic outcomes, which were found to show a more accurate prediction in risk stratification for TNBC patients. Moreover, the splicing correlation network between prognostic splicing factors and aberrant AS events revealed a strong regulatory mode, which could provide a better understanding of the underlying mechanisms in the TNBC spliceosome. The work achieved in our study could not only contribute to elucidating the tumorigenic mechanism of AS, it could also provide new clues to uncover potential prognostic biomarkers and therapeutic targets for further study.

## Data Availability Statement

RNAseq-HTSeq data and clinical data of patients who were diagnosed with primary breast cancer were downloaded from the TCGA (https://tcga-data.nci.nih.gov/tcga/). AS profiling of TNBC cohorts were downloaded from TCGA SpliceSeq (http://bioinformatics.mdanderson.org/TCGASpliceSeq).

## Author Contributions

SG and ZS designed the study and wrote the original draft. All authors analyzed and reviewed the data, read and agreed to the published version of the manuscript. SG, ZS, and DS-L edited the manuscript.

## Conflict of Interest

The authors declare that the research was conducted in the absence of any commercial or financial relationships that could be construed as a potential conflict of interest.

## Footnotes

1 https://tcga-data.nci.nih.gov/tcga/

2 http://bioinformatics.mdanderson.org/TCGASpliceSeq

3 http://software.broadinstitute.org/gsea/index.jsp

4 www.introni.it/spliceaid.html

## Abbreviations

AA: alternate acceptor site
AD: alternate donor site
AP: alternate promoter
AS: alternative splicing
AT: alternate terminator
AUC: area under the curve
BP: biological process
CC: cellular component
DAVID: Database for Annotation Visualization and Integrated Discovery
ES: exon skip
GO: gene ontology
GSEA: gene set enrichment analysis
HR: hazard ratios
KEGG: Kyoto Encyclopedia of Genes and Genomes
ME: mutually exclusive exons
MF: molecular function
MSigDB: Molecular Signatures Database
OS: overall survival
PSI: percent spliced in
RI: retained intron
ROC: receiver operator characteristic
SF: splicing factor
TCGA: The Cancer Genome Atlas
TNBC: triple-negative breast cancer.
